# Supplementary material for: Functional disease architectures reveal unique biological role of transposable elements
Source: Nat Commun. 2019 Sep 6;10:4054. doi: 10.1038/s41467-019-11957-5 (PMC6731302; doi:10.1038/s41467-019-11957-5)
Supplement: Supplementary file 1 — Supplementary Information [file 41467_2019_11957_MOESM1_ESM.pdf]

Supplementary Tables

Annotation	#Source	Model
base	Finucane et al.[1]	baseline/baselineLD
Coding	UCSC Genome Browser and Finucane et al.[1]	baseline/baselineLD
3' UTR	UCSC Genome Browser and Finucane et al.[1]	baseline/baselineLD
5' UTR	UCSC Genome Browser and Finucane et al.[1]	baseline/baselineLD
Promoter	UCSC Genome Browser and Finucane et al.[1]	baseline/baselineLD
Intron	UCSC Genome Browser and Finucane et al.[1]	baseline/baselineLD
ENCODE TF binding	ENCODE[2] and Finucane et al.[1]	baseline/baselineLD
CTCF promoter	Hoffman et al[3] and Finucane et al.[1]	baseline/baselineLD
Promotor-Flanking	Hoffman et al[3] and Finucane et al.[1]	baseline/baselineLD
Transcribed	Hoffman et al[3] and Finucane et al.[1]	baseline/baselineLD
Transcription start size (TSS)	Hoffman et al[3] and Finucane et al.[1]	baseline/baselineLD
Strong enhancer	Hoffman et al[3] and Finucane et al.[1]	baseline/baselineLD
Weak enhancer	Hoffman et al[3] and Finucane et al.[1]	baseline/baselineLD
DHS	ENCODE[2]/Roadmap[4] post-processed by Trynka et al and Finucane et al.[1]	baseline/baselineLD
H3K4me1	Roadmap Epigenomics[4] post-processed by Trynka et al[5] and Finucane et al.[1]	baseline/baselineLD
H3K4me3	Roadmap Epigenomics[4] post-processed by Trynka et al[5] and Finucane et al.[1]	baseline/baselineLD
H3K9ac	Roadmap Epigenomics[4] post-processed by Trynka et al[5] and Finucane et al.[1]	baseline/baselineLD
H3K27ac	Roadmap Epigenomics[4] post-processed by Trynka et al[5] and Finucane et al.[1]	baseline/baselineLD
H3K27ac	Hnisz et al[6] and Finucane et al.[1]	baseline/baselineLD
Super Enhancer	Hnisz et al[6] and Finucane et al.[1]	baseline/baselineLD
Conserved	Lindblad-Toh et al.[7] and Finucane et al.[1]	baseline/baselineLD
FANTOM5 enhancer	Anderson et al.[8] and Finucane et al.[1]	baseline/baselineLD
2 GERP[9] conserved	Gazal et al.[10]	baselineLD
10 MAF bin	Gazal et al.[10]	baselineLD
Predicted Allele Age	Gazal et al.[10]	baselineLD
LLD-AFR	Gazal et al.[10]	baselineLD
Recombination Rate	Gazal et al.[10]	baselineLD
Nucleotide Diversity	Gazal et al.[10]	baselineLD
Background Selection Statistics	Gazal et al.[10]	baselineLD
CpG-Content	Gazal et al.[10]	baselineLD

Supplementary Table 1. List of 75 functional and LD related annotations in the baselineLD model (v1.1). There are 24 annotations in the baselineLD where we add a 500bp window around the category. In the case of DHS, H3K4me1, H3K4me3, and H3K27ac annotations we add 100-bp window.

TE	Expected (%SNPs)	$\tau * (se)$	$\tau*$ (P)	Enrichment (se)	Enrichment (P)	Observed	Expected (baseline-LD)
ALLTE	53.81	0.31 (0.05)	1.14E-11	0.72 (0.03)	3.68E-12	38.52	18.42
LINE	21.41	0.18 (0.05)	1.73E-04	0.73 (0.05)	4.66E-07	15.57	4.78
SINE	16.32	0.43 (0.06)	3.09E-12	1.18 (0.11)	2.73E-01	19.25	6.05
LTR	9.88	0.14 (0.03)	2.90E-05	0.38 (0.07)	2.81E-08	3.76	2.02
DNA	3.28	0.15 (0.04)	8.64E-04	1.23 (0.19)	6.68E-01	4.04	4.03

Supplementary Table 2. S-LDSC results for four main TE classes and ALLTE. We compute the heritability enrichment and $\tau*$ for four main TE classes (LINE, SINE, LTR, and DNA) and ALLTE (combination of all TE) conditional on baseline-LD model. These results are obtained by meta-analyzing 41 independent traits and disease.

TE	Enrichment (Farh et al.)	Enrichment (Huang et al.)	S-LDSC
ALLTE	0.81 (0.01)	0.92 (0.03)	0.72(0.03)
LINE	0.74 (0.03)	0.79 (0.08)	0.73(0.05)
SINE	1.08 (0.04)	1.23 (0.13)	1.18(0.11)
LTR	0.64 (0.03)	0.77 (0.11)	0.38(0.07)
DNA	1.01 (0.09)	1.07 (0.16)	1.23(0.19)

Supplementary Table 3. Enrichment of fine-mapped causal disease SNPs for four main TE classes and ALLTE. We computed the enrichment of fine-mapped causal disease SNPs as the proportion of fine-mapped SNPs (from Farh et al.[14] and Huang et al.[15], for autoimmune diseases) that line in TE divide by the proportion of common variants that lie in TE. This is a conservative computation (results may be biased towards 1) because fine-mapped SNPs were defined based on 95% causal sets and may include SNPs that are not actually causal. We also report the disease heritability enrichment estimated using S-LDSC, meta-analyzed across 41 independent traits (47 traits).

TE	Enrichment (fine-mapping) FE-Meta-Tissue	Enrichment (fine-mapping) Whole Blood	S-LDSC
ALLTE	0.89 (0.01)	0.88 (0.01)	0.72(0.03)
LINE	0.67 (0.01)	0.61 (0.01)	0.73(0.05)
SINE	1.30 (0.02)	1.34 (0.02)	1.18(0.11)
LTR	0.78 (0.01)	0.78 (0.01)	0.38(0.07)
DNA	0.87 (0.02)	0.82 (0.03)	1.23(0.19)

Supplementary Table 4. Enrichment of fine-mapped causal cis-eQTL SNPs for four main TE classes and ALLTE. We computed the enrichment of fine-mapped causal cis-eQTL SNPs as the average MaxCPP (maximum Causal Posterior Probability) score[13] of variants that lie in TE divided by the average MaxCPP score of all common SNPs. We obtained MaxCPP scores for FE-Meta-Tissue and Whole blood from ref.[13]. We also report the disease heritability enrichment estimated using S-LDSC, meta-analyzed across 41 independent traits (47 traits); identical to Supplementary Table 3.

TE	Expected (%SNPs)	$\tau*(se)$	$\tau*$ (P)	Enrichment (se)	Enrichment (P)
LINE	21.41	0.25 (0.04)	2.19E-08	0.67 (0.04)	3.63E-08
SINE	16.32	0.48 (0.07)	4.57E-12	1.13 (0.11)	4.67E-01
LTR	9.88	0.21 (0.03)	8.84E-10	0.35 (0.07)	8.80E-08
DNA	3.28	0.20 (0.04)	2.21E-07	1.20 (0.18)	5.93E-01

Supplementary Table 5. S-LDSC results for joint analysis of four main TE classes. We compute the heritability enrichment and $\tau*$ for four main TE classes (LINE, SINE, LTR, and DNA) conditional on each other and baseline-LD model. These results are obtained by meta-analyzing 41 independent traits and disease.

Quintile	Expected (baseline-LD)	Observed Enrichment (se)	Observed $\tau*(se)$
Q1	0.01 (0.04)	0.37 (0.10)	-0.02 (0.03)
Q2	0.17 (0.05)	0.63 (0.09)	0.05 (0.03)
Q3	0.24 (0.04)	0.65 (0.06)	0.01 (0.03)
Q4	0.50 (0.02)	0.99 (0.08)	0.05 (0.03)
Q5	0.77 (0.02)	0.91 (0.11)	-0.10 (0.04)

Supplementary Table 6. S-LDSC results for all TE in different age quintiles. We report the Expected (baseline-LD) enrichment and Observed enrichment and $\tau*$ of SNPs in ALLTE for five different age quintiles where Q1 is the youngest and Q5 is the oldest. We computed the Observed enrichment and $\tau*$ conditional on ALLTE and the baseline-LD model. Error bars represent 95% confidence intervals.

TE	Expected (%SNP)	$\tau*(se)$	$\tau*$ (P)	Enrichment (se)	Enrichment (P)
ALU	13.59	0.49(0.06)	1.12E-15	1.18(0.12)	2.35E-01
AluJb	1.19	-0.01(0.05)	8.39E-01	0.79(0.35)	1.26E-01
AluJo	0.58	0.02(0.05)	6.12E-01	1.38(0.52)	5.41E-01
AluJr	0.65	0.00(0.05)	9.67E-01	1.14(0.62)	9.53E-01
AluSc	0.47	0.02(0.05)	7.23E-01	0.63(0.54)	9.75E-03
AluSg	0.56	-0.06(0.05)	2.05E-01	-0.36(0.56)	1.65E-01
AluSp	0.66	0.13(0.06)	3.02E-02	1.76(0.60)	1.36E-01
AluSq2	0.70	-0.01(0.06)	8.02E-01	0.59(0.56)	9.20E-01
AluSx	1.53	0.05(0.05)	3.23E-01	0.92(0.37)	9.11E-01
AluSx1	1.37	0.06(0.05)	1.84E-01	1.00(0.30)	2.20E-01
AluSz	1.15	0.04(0.05)	3.35E-01	1.01(0.34)	9.20E-01
AluSz6	0.47	0.02(0.05)	7.14E-01	0.63(0.54)	8.03E-02
AluY	2.22	-0.03(0.06)	6.45E-01	0.18(0.29)	5.68E-03
**ERV1**	3.35	0.16(0.03)	2.11E-06	0.37(0.09)	9.08E-07
ERVL	5.98	0.16(0.04)	2.09E-04	0.62(0.11)	2.82E-02
ERVL-MaLR	3.96	0.09(0.04)	3.39E-02	0.43(0.16)	9.39E-03
hAT-Charlie	1.50	0.10(0.04)	1.66E-02	1.21(0.33)	9.66E-01
**L1**	17.56	0.16(0.04)	3.23E-04	0.62(0.05)	2.18E-10
L1M5	0.53	-0.02(0.04)	6.20E-01	-0.06(0.48)	1.25E-01
L1ME1	0.49	0.04(0.05)	3.96E-01	1.16(0.40)	9.73E-01
**L1PA3**	0.93	0.04(0.05)	4.93E-01	-0.04(0.16)	3.57E-08
**L1PA4**	0.94	-0.05(0.05)	3.43E-01	0.13(0.14)	2.39E-04
L1PA5	0.75	0.11(0.05)	2.42E-02	0.49(0.16)	7.48E-03
L1PA6	0.45	0.10(0.06)	1.17E-01	0.68(0.35)	2.02E-01
L1PA7	0.71	0.10(0.05)	6.35E-02	0.92(0.20)	3.50E-01
L1PB1	0.44	0.09(0.03)	1.32E-02	0.48(0.21)	1.16E-01
L2	0.49	-0.07(0.04)	8.20E-02	-0.01(0.44)	9.73E-02
L2-family	3.41	-0.01(0.04)	7.53E-01	0.81(0.17)	5.37E-01
L2a	1.42	0.02(0.04)	6.90E-01	0.89(0.28)	2.71E-01
L2b	0.62	0.03(0.04)	4.62E-01	1.30(0.47)	8.49E-01
L2c	0.88	-0.01(0.04)	8.08E-01	0.54(0.35)	9.28E-02
MIR	0.89	0.02(0.05)	7.32E-01	1.01(0.46)	9.57E-01
MIRb	1.10	-0.01(0.04)	7.51E-01	0.96(0.37)	4.98E-01
MIRc	0.41	0.03(0.04)	3.82E-01	1.92(0.56)	1.91E-01
TcMar-Tigger	1.13	0.15(0.04)	1.52E-04	1.37(0.26)	6.17E-01

Supplementary Table 7. S-LDSC results for 35 TE families/subfamilies spanning at least 0.4% of common SNPs. We applied S-LDSC to 35 TE that capture at least 0.4% of common SNPs. These results are obtained by meta-analyzing 41 independent traits and disease conditional on baselineLD model. TE families/subfamilies that are significantly depleted for heritability (enrichment < 1) after correcting for 35 hypotheses tested ($P < 0.05/35$) are indicated in bold font.

TE	Expected (%SNP)	$\tau * (se)$	$\tau*$ (P)	Enrichment (se)	Enrichment (P)
L1	17.56	0.16 (0.04)	3.23E-04	0.62 (0.05)	2.18E-10
L1PA3	0.93	0.04 (0.05)	4.93E-01	-0.04 (0.16)	3.57E-08
ERV1	3.35	0.16 (0.03)	2.11E-06	0.37 (0.09)	9.08E-07
L1PA4	0.94	-0.05 (0.05)	3.43E-01	0.13 (0.14)	2.39E-04

Supplementary Table 8. List of TE families/subfamilies spanning at least 0.4% of common SNPs with significant enrichment conditional on baseline-LD model. We applied S-LDSC to 35 families/subfamilies spanning at least 0.4% of SNPs, to compute the enrichment and $\tau*$ of each TE meta-analyzed across 41 independent traits (47 traits) in a joint model that consist of TE and baseline-LD model (TE+baseline-LD). We reported the set of TE that have significant enrichment conditional on baseline-LD model after correcting for the number of multiple hypothesis tests ($P < \frac{0.05}{35}$).

TE	Enrichment (se)	Enrichment (P)
AmnSINE1	5.44 (0.32)	1.40E-64
Charlie13a	1.36 (0.07)	2.12E-05
Charlie15a	1.23 (0.04)	1.01E-07
Charlie19a	1.44 (0.04)	9.10E-14
Charlie20a	1.27 (0.06)	4.79E-06
Charlie26a	1.43 (0.05)	7.53E-08
CR1-Hg19	1.33 (0.05)	2.64E-32
CR1-Mam	1.97 (0.11)	1.48E-31
L3	1.11 (0.04)	2.79E-08
L3b	1.81 (0.07)	5.33E-41
L5	1.71 (0.07)	3.11E-17
LFSINE-Vert	5.54 (0.39)	3.05E-72
LTR10B1	2.08 (0.34)	4.91E-09
LTR14	2.09 (0.26)	1.14E-05
LTR90A	1.65 (0.08)	1.69E-09
LTR90B	1.36 (0.12)	5.66E-05
Mam-R4	2.67 (0.18)	3.78E-33
MamRep1161	1.28 (0.05)	5.61E-08
MamRep434	2.33 (0.13)	1.78E-41
MamSINE1	2.20 (0.13)	9.11E-32
MARNA	1.29 (0.05)	9.27E-11
MER102b	1.19 (0.04)	9.13E-08
MER102c	1.15 (0.04)	2.75E-05
MER117	1.54 (0.06)	1.16E-29
MER121	6.21 (0.46)	1.47E-76
MER135	3.60 (0.32)	9.12E-35
MER81	1.14 (0.03)	4.12E-05
MER91A	1.32 (0.05)	4.45E-13
MER94B	1.57 (0.08)	2.64E-07
MIR3	1.42 (0.03)	1.99E-55
MLT1K	1.18 (0.05)	3.66E-09
MLT1M	1.42 (0.05)	4.01E-18
Plat-L3	2.77 (0.15)	1.83E-60
Tigger10	1.30 (0.06)	1.05E-05
Tigger12	1.45 (0.06)	1.16E-06
Tigger12A	1.40 (0.05)	4.24E-08
Tigger12c	1.54 (0.06)	1.40E-15
Tigger14a	1.70 (0.07)	5.95E-20
Tigger15a	1.44 (0.04)	3.98E-27
Tigger16b	1.55 (0.08)	5.06E-12
UCON29	5.58 (0.42)	4.49E-51
X3-LINE	2.31 (0.17)	4.61E-19
X6B-LINE	4.93 (0.38)	3.66E-43
X7A-LINE	1.24 (0.04)	1.44E-06
X7C-LINE	1.83 (0.07)	2.33E-17
X8-LINE	3.57 (0.28)	2.75E-30

Supplementary Table 9. Set of TE families/subfamilies that are enriched for the Expected (baseline-LD) heritability of disease and complex traits.

**Trait Group**	**Trait**	Source	*N*
Blood	Platelet Count	UKBiobank	444,382
	Red Blood Cell Count	UKBiobank	445,174
	Red Blood Cell Distribution Width	UKBiobank	442,700
	Eosinophil Count	UKBiobank	439,938
	White Blood Cell Count	UKBiobank	444,502
Autoimmune	Crohn's Disease	Jostins et al., 2012 Nature	20,883
	Rheumatoid Arthritis	Okada et al., 2014 Nature	37,681
	Ulcerative Colitis	Jostins et al., 2012 Nature	27,432
	Celiac	Dubois et al., 2010 Nat Genet	15,283
	Lupus	Bentham et al., 2015 Nat Genet	14,267
	Auto Immune Traits (Sure)	UKBiobank	459,324
Brain	Age at Menarche	UKBiobank	242,278
	BMI	Speliotes et al., 2010 Nat Genet	122,033
	BMI	UKBiobank	457,824
	Bipolar Disorder	BIP Working Group, 2011 Nat Genet	16,731
	Depressive symptoms	Okbay et al., 2016 Nat Genet	161,460
	Neuroticism	UKBiobank	372,066
	Schizophrenia	SCZ Working Group, 2014 Nature	70,100
	Smoking Status	TAG Consortium, 2010 Nat Genet	74,035
	Smoking Status	UKBiobank	457,683
	Year of education	UKBiobank	328,917
	Year of education	Rietveld et al., 2013 Science	126,559

Supplementary Table 10. List of blood, autoimmune and brain-related diseases and complex traits. We analyzed 5 blood traits, 6 autoimmune diseases, and 8 brain-related traits. For some traits, we analyzed more than one data set.

**TE**	**Excess Blood (se)**	**Excess Other (se)**
ALLTE	0.70 (0.010)	0.67 (0.010)
LINE	0.48 (0.005)	0.49 (0.004)
SINE	0.90 (0.011)	0.72 (0.007)
LTR	0.75 (0.011)	0.90 (0.008)
DNA	0.90 (0.010)	0.94 (0.008)

Supplementary Table 12. Excess overlap of each TE class with blood chromatin data and other chromatin data. Excess overlap is computed by computing the overlap of SINE and blood active chromatin mark and non-blood active chromatin mark. We obtained the blood active chromatin regions by combining 27 blood cells and 6 chromatin mark (H3K27ac, H3K4me3, DNase, DNase-H3K27ac, DNase-H3K4me3) obtained from ChromImpute[16] applied on RoadMap[4] and we obtained the non-blood active chromatin regions by combining 100 non-blood cells and 6 chromatin mark.

**Trait(T)**	**TE**	**Enr. (se)** Trait T	**$\tau*$ T (se)**	**Enr. other (se)**	**$\tau*$ other (se)**	**Expected Enr.** Trait T (se)	**Expected Enr.** other traits
Blood	ALLTE	0.80(0.11)	0.37(0.14)	0.68(0.03)	0.28(0.05)	0.85 (0.02)	0.84 (0.01)
Blood	LINE	0.75(0.13)	0.27(0.09)	0.72(0.06)	0.16(0.05)	0.55 (0.03)	0.57 (0.02)
Blood	SINE	2.05(0.30)	0.88(0.14)	0.96(0.09)	0.33(0.06)	1.05 (0.04)	0.93 (0.01)
Blood	LTR	0.35(0.16)	0.24(0.13)	0.38(0.08)	0.13(0.04)	0.44 (0.07)	0.53 (0.02)
Blood	DNA	0.80(0.41)	0.10(0.10)	1.26(0.23)	0.14(0.05)	0.80 (0.03)	0.87 (0.01)
Autoimmune	ALLTE	0.96(0.17)	0.67(0.29)	0.68(0.03)	0.28(0.05)	0.85 (0.02)	0.84 (0.01)
Autoimmune	LINE	1.28(0.36)	0.70(0.30)	0.72(0.06)	0.16(0.05)	0.50 (0.07)	0.57 (0.01)
Autoimmune	SINE	1.33(0.69)	0.47(0.42)	0.96(0.09)	0.33(0.06)	1.09 (0.04)	0.93 (0.01)
Autoimmune	LTR	0.77(0.80)	0.44(0.40)	0.38(0.08)	0.13(0.04)	0.56 (0.04)	0.52 (0.02)
Autoimmune	DNA	2.97(2.38)	0.60(0.55)	1.26(0.23)	0.14(0.05)	0.81 (0.05)	0.86 (0.01)
Brain	ALLTE	0.64(0.04)	0.21(0.06)	0.69(0.03)	0.29(0.05)	0.84 (0.01)	0.84 (0.01)
Brain	LINE	0.63(0.10)	0.09(0.07)	0.71(0.06)	0.16(0.05)	0.64 (0.02)	0.55 (0.02)
Brain	SINE	0.86(0.11)	0.25(0.07)	1.00(0.09)	0.35(0.06)	0.87 (0.02)	0.96 (0.01)
Brain	LTR	0.73(0.14)	0.24(0.06)	0.39(0.09)	0.14(0.04)	0.42 (0.02)	0.55 (0.02)
Brain	DNA	1.32(0.28)	0.16(0.07)	1.29(0.23)	0.15(0.05)	0.90 (0.01)	0.85 (0.01)

Supplementary Table 11. Trait class-specific S-LDSC results for each TE class. In the case of blood and autoimmune traits the expected enrichment is computed using baseline-LD and blood chromatin annotation (Expected (baeline-LD+blood chromatin)) and for brain-related traits the expected enrichment is computed using baseline-LD and brain chromatin annotation (Expected (baeline-LD+brain chromatin)). Heritability and $\tau*$ are meta-analyzed over subset of traits.

**TE**	Expected (%SNPs)	**Enr Blood (se)**	**Enr Other (se)**	$\tau*$ **Blood (se)**	$\tau*$ **Other (se)**	**Excess Blood (se)**	**Excess Other (se)**
GCGGTGGCT	0.5%	6.13 (1.59)	-0.64 (0.69)	0.35 (0.11)	-0.09 (0.05)	1.05 (0.01)	0.86 (0.01)
GTGGTGGCT	0.6%	6.65 (1.37)	-0.70 (0.58)	0.48 (0.11)	-0.11 (0.04)	0.98 (0.01)	0.81 (0.01)

Supplementary Table 13. Higher enrichment of GCGGTGGCT and GTGGTGGCT for blood traits. We create an annotation for each 9-mer by detecting regions of the genome that map the 9-mer with at most one mismatch using mrsFAST[17]. mrsFAST is a fully sensitive mapper that guarantees to detect all possible mapping position for a given sequence. Excess overlap is computed by computing the overlap of an annotation and blood active chromatin mark and non-blood active chromatin mark. We obtained the blood active chromatin regions by combining 27 blood cells and 6 chromatin mark (H3K27ac, H3K4me3, DNase, DNase-H3K27ac, DNase-H3K4me3) obtained from ChromImpute[16] applied on RoadMap[4] and we obtained the non-blood active chromatin regions by combining 100 non-blood cells and 6 chromatin mark.

**TE**	**Excess Blood (se)**	**Excess Other (se)**
GCGGTGGCT-ALU	0.85 (0.01)	0.63 (0.01)
GCGGTGGCT-NONALU	1.67 (0.03)	1.59 (0.02)
GTGGTGGCT-ALU	0.80 (0.01)	0.57 (0.01)
GTGGTGGCT-NONALU	1.39 (0.02)	1.37 (0.01)

Supplementary Table 14. Excess overlap of GCGGTGGCT and GTGGTGGCT (both ALU and NONALU) with blood chromatin and other chromatin. ALU denotes regions of the genome that lie inside an Alu TE. NONALU denotes regions of the genome that do not lie inside an Alu TE. Excess overlap is computed by computing the overlap of an annotation and blood active chromatin mark and non-blood active chromatin mark. We obtained the blood active chromatin regions by combining 27 blood cells and 6 chromatin mark (H3K27ac, H3K4me3, DNase, DNase-H3K27ac, DNase-H3K4me3) obtained from ChromImpute[16] applied on RoadMap[4] and we obtained the non-blood active chromatin regions by combining 100 non-blood cells and 6 chromatin mark.

**TE**	**Enrichment Blood (se)**	**Enrichment other (se)**
ALU	2.01 (0.25)	0.97 (0.11)
AluJb	1.22 (1.12)	0.65 (0.37)
AluJo	2.60 (1.44)	1.12 (0.62)
AluJr	2.11 (1.28)	0.92 (0.75)
AluSc	1.39 (1.67)	0.47 (0.57)
AluSg	1.70 (1.29)	-0.80 (0.64)
AluSp	4.25 (2.06)	1.20 (0.58)
AluSq2	0.28 (1.28)	0.65 (0.64)
AluSx1	1.19 (0.83)	0.89 (0.33)
AluSx	1.34 (1.37)	0.74 (0.35)
AluSz6	-2.55 (2.19)	0.66 (0.67)
AluSz	1.19 (0.90)	0.94 (0.37)
AluY	0.15 (0.59)	0.17 (0.34)
DNA	0.89 (0.41)	1.26 (0.23)
ERV1	0.16 (0.22)	0.42 (0.10)
ERVL	0.62 (0.22)	0.59 (0.13)
ERVL-MaLR	0.03 (0.28)	0.51 (0.18)
hAT-Charlie	0.46 (0.64)	1.37 (0.38)
L1	0.76 (0.10)	0.61 (0.06)
L1M5	-0.90 (1.27)	0.35 (0.49)
L1ME1	0.98 (0.83)	1.30 (0.48)
L1PA3	0.07 (0.32)	-0.04 (0.18)
L1PA4	0.49 (0.30)	0.07 (0.17)
L1PA5	-0.38 (0.33)	0.74 (0.19)
L1PA6	1.02 (0.72)	0.59 (0.40)
L1PA7	0.95 (0.48)	0.89 (0.22)
L1PB1	0.63 (0.48)	0.46 (0.24)
L2a	0.69 (0.67)	0.95 (0.31)
L2b	-0.03 (1.80)	1.70 (0.44)
L2-family	1.19 (1.00)	-0.39 (0.49)
L2c	-0.57 (0.87)	0.77 (0.39)
L2	0.61 (0.53)	0.86 (0.18)
LINE	0.81 (0.12)	0.72 (0.06)
LTR	0.39 (0.17)	0.38 (0.08)
MIRb	1.95 (1.03)	0.86 (0.40)
MIR	2.79 (1.21)	0.79 (0.49)
MIRc	0.94 (1.38)	2.13 (0.61)
ALLTE	0.83 (0.09)	0.68 (0.03)
SINE	1.96 (0.24)	0.96 (0.09)
TcMar-Tigger	1.67 (0.72)	1.30 (0.29)

Supplementary Table 15. Comparison of Observed heritability enrichment of 35 TE family/subfamilies spanning at least 0.4% of common SNPs for blood traits and other traits.

TE	Enrichment Autoimmune (se)	Enrichment other (se)
ALU	2.01 (0.25)	0.97 (0.11)
AluJb	1.22 (1.12)	0.65 (0.37)
AluJo	2.60 (1.44)	1.12 (0.62)
AluJr	2.11 (1.28)	0.92 (0.75)
AluSc	1.39 (1.67)	0.47 (0.57)
AluSg	1.70 (1.29)	-0.80 (0.64)
AluSp	4.25 (2.06)	1.20 (0.58)
AluSq2	0.28 (1.28)	0.65 (0.64)
AluSx1	1.19 (0.83)	0.89 (0.33)
AluSx	1.34 (1.37)	0.74 (0.35)
AluSz6	-2.55 (2.19)	0.66 (0.67)
AluSz	1.19 (0.90)	0.94 (0.37)
AluY	0.15 (0.59)	0.17 (0.34)
DNA	0.89 (0.41)	1.26 (0.23)
ERV1	0.16 (0.22)	0.42 (0.10)
ERVL	0.62 (0.22)	0.59 (0.13)
ERVL-MaLR	0.03 (0.28)	0.51 (0.18)
hAT-Charlie	0.46 (0.64)	1.37 (0.38)
L1	0.76 (0.10)	0.61 (0.06)
L1M5	-0.90 (1.27)	0.35 (0.49)
L1ME1	0.98 (0.83)	1.30 (0.48)
L1PA3	0.07 (0.32)	-0.04 (0.18)
L1PA4	0.49 (0.30)	0.07 (0.17)
L1PA5	-0.38 (0.33)	0.74 (0.19)
L1PA6	1.02 (0.72)	0.59 (0.40)
L1PA7	0.95 (0.48)	0.89 (0.22)
L1PB1	0.63 (0.48)	0.46 (0.24)
L2a	0.69 (0.67)	0.95 (0.31)
L2b	-0.03 (1.80)	1.70 (0.44)
L2-family	1.19 (1.00)	-0.39 (0.49)
L2c	-0.57 (0.87)	0.77 (0.39)
L2	0.61 (0.53)	0.86 (0.18)
LINE	0.81 (0.12)	0.72 (0.06)
LTR	0.39 (0.17)	0.38 (0.08)
MIRb	1.95 (1.03)	0.86 (0.40)
MIR	2.79 (1.21)	0.79 (0.49)
MIRc	0.94 (1.38)	2.13 (0.61)
ALLTE	0.83 (0.09)	0.68 (0.03)
SINE	1.96 (0.24)	0.96 (0.09)
TcMar-Tigger	1.67 (0.72)	1.30 (0.29)

Supplementary Table 16. Comparison of Observed heritability enrichment of 35 TE families/subfamilies spanning at least 0.4% of common SNPs for autoimmune traits and other traits

**TE**	**Enrichment Brain traits(se)**	**Enrichment other traits(se)**
ALU	0.82 (0.15)	1.00 (0.10)
AluJb	0.97 (0.55)	0.71 (0.37)
AluJo	1.83 (0.86)	1.12 (0.62)
AluJr	1.77 (0.84)	0.92 (0.74)
AluSc	1.63 (0.88)	0.44 (0.57)
AluSg	0.03 (1.04)	-0.76 (0.64)
AluSp	0.06 (0.75)	1.28 (0.58)
AluSq2	-1.00 (0.76)	0.71 (0.64)
AluSx1	0.48 (0.50)	0.96 (0.33)
AluSx	1.12 (0.50)	0.76 (0.36)
AluSz6	0.77 (0.90)	0.83 (0.59)
AluSz	0.27 (0.66)	0.96 (0.37)
AluY	-1.07 (0.35)	0.19 (0.34)
DNA	1.32 (0.28)	1.29 (0.23)
ERV1	0.73 (0.15)	0.45 (0.10)
ERVL	0.66 (0.26)	0.58 (0.13)
ERVL-MaLR	0.32 (0.30)	0.50 (0.18)
hAT-Charlie	0.94 (0.65)	1.39 (0.37)
L1	0.55 (0.12)	0.60 (0.06)
L1M5	1.05 (1.02)	0.32 (0.48)
L1ME1	0.48 (0.98)	1.26 (0.44)
L1PA3	-0.01 (0.30)	-0.04 (0.18)
L1PA4	-0.40 (0.33)	0.06 (0.17)
L1PA5	0.73 (0.28)	0.76 (0.19)
L1PA6	-0.57 (0.68)	0.59 (0.40)
L1PA7	0.90 (0.34)	0.91 (0.22)
L1PB1	0.19 (0.40)	0.48 (0.24)
L2a	1.08 (0.64)	0.93 (0.31)
L2b	2.01 (0.73)	1.70 (0.44)
L2	-0.62 (0.81)	-0.37 (0.48)
L2c	0.97 (0.71)	0.78 (0.39)
L2-family	0.93 (0.41)	0.85 (0.18)
LINE	0.63 (0.10)	0.71 (0.06)
LTR	0.73 (0.14)	0.39 (0.09)
MIRb	2.04 (0.70)	1.12 (0.47)
MIR	1.76 (0.62)	0.76 (0.49)
MIRc	3.57 (0.98)	2.11 (0.61)
ALLTE	0.64 (0.04)	0.69 (0.03)
SINE	0.86 (0.11)	1.00 (0.09)
TcMar-Tigger	2.12 (0.45)	1.33 (0.29)

Supplementary Table 17. Comparison of Observed heritability enrichment of 35 TE families/subfamilies spanning at least 0.4% of common SNPs for brain traits and other traits.

TE	Repeat Class	Expected Enr. Blood traits(se)	Expected Enr other traits(se)	Enr P Blood vs. other traits
FLAM-C	SINE	1.49 (0.11)	1.02 (0.04)	1.43E-05
FRAM	SINE	1.38 (0.09)	0.94 (0.04)	6.71E-06
Harlequin-int	LTR	0.82 (0.09)	0.35 (0.07)	2.48E-05
HERV15-int	LTR	2.52 (0.34)	0.73 (0.15)	8.33E-07
HERV3-int	LTR	1.58 (0.19)	0.50 (0.14)	1.49E-06
HERVFH21-int	LTR	1.25 (0.28)	-0.01 (0.13)	2.56E-05
LTR13A	LTR	2.50 (0.26)	1.34 (0.13)	4.05E-05
LTR14	LTR	2.88 (0.34)	1.18 (0.16)	3.98E-06
LTR15	LTR	2.94 (0.33)	0.97 (0.12)	7.53E-09
LTR19C	LTR	2.15 (0.22)	0.86 (0.13)	2.03E-07
LTR21A	LTR	2.26 (0.27)	0.76 (0.15)	7.82E-07
LTR24B	LTR	1.44 (0.13)	0.67 (0.09)	4.34E-07
LTR2B	LTR	2.58 (0.22)	1.10 (0.15)	1.48E-08
LTR2C	LTR	1.84 (0.22)	0.85 (0.10)	1.87E-05
LTR46	LTR	1.60 (0.16)	0.53 (0.10)	5.61E-09
LTR4	LTR	3.98 (0.47)	1.57 (0.27)	3.99E-06
LTR54	LTR	1.25 (0.09)	0.76 (0.05)	2.06E-06
LTR61	LTR	1.53 (0.25)	0.39 (0.12)	2.04E-05
LTR62	LTR	1.54 (0.17)	0.61 (0.10)	1.03E-06
LTR65	LTR	1.48 (0.11)	0.91 (0.04)	8.95E-07
LTR75-1	LTR	1.30 (0.18)	0.48 (0.06)	7.68E-06
LTR7C	LTR	1.45 (0.13)	0.69 (0.12)	5.30E-06
MER34C2	LTR	1.37 (0.12)	0.81 (0.05)	1.26E-05
MER51B	LTR	1.06 (0.08)	0.60 (0.07)	3.95E-06
MER51D	LTR	1.53 (0.17)	0.39 (0.10)	5.54E-09
MER57E3	LTR	5.19 (0.47)	1.00 (0.26)	4.75E-15
MER65C	LTR	0.98 (0.09)	0.56 (0.06)	2.04E-05

Supplementary Table 18. Comparison of Expected (baseline-LD+blood chromatin) enrichment of TE families/subfamilies in blood cell-type traits and non-blood cell-type traits. These are TE families/subfamilies that are significantly different enriched between blood and non-blood traits.

TE	Repeat Class	Expected Enr. Autoimmune traits(se)	Expected Enr other traits(se)	Enr P Autoimmune vs. other traits
FLAM-C	SINE	1.54 (0.11)	1.04 (0.04)	1.38E-05
FRAM	SINE	1.28 (0.06)	0.97 (0.04)	1.83E-05
HERVFH21-int	LTR	4.14 (0.84)	0.03 (0.12)	6.53E-07
LOR1a	LTR	1.13 (0.12)	0.52 (0.05)	1.71E-06
LTR10A	LTR	2.77 (0.37)	0.90 (0.13)	1.14E-06
LTR10B	LTR	2.80 (0.40)	0.81 (0.14)	1.27E-06
LTR10F	LTR	1.50 (0.23)	0.48 (0.11)	3.82E-05
LTR12F	LTR	1.65 (0.29)	0.34 (0.06)	3.49E-06
LTR13A	LTR	3.36 (0.44)	1.38 (0.13)	7.18E-06
LTR19A	LTR	1.67 (0.25)	0.61 (0.10)	3.85E-05
LTR21A	LTR	4.12 (0.76)	0.84 (0.15)	1.11E-05
LTR2	LTR	1.61 (0.16)	0.79 (0.08)	2.66E-06
LTR4	LTR	7.54 (1.09)	1.62 (0.26)	6.66E-08
LTR62	LTR	1.71 (0.21)	0.65 (0.10)	3.82E-06
LTR76	LTR	3.06 (0.49)	1.00 (0.17)	3.65E-05
LTR7C	LTR	1.85 (0.26)	0.72 (0.12)	4.06E-05
MER41B	LTR	1.70 (0.10)	0.93 (0.08)	1.54E-09
MER41D	LTR	1.55 (0.21)	0.55 (0.07)	1.91E-06
MER41E	LTR	1.96 (0.27)	0.65 (0.08)	1.84E-06
MER51D	LTR	2.22 (0.33)	0.45 (0.11)	2.09E-07
MER57F	LTR	1.88 (0.32)	0.54 (0.06)	2.40E-05
MER83B	LTR	2.67 (0.42)	0.96 (0.09)	3.82E-05
MLT1F	LTR	1.35 (0.07)	0.76 (0.03)	2.40E-13
MLT1G3	LTR	1.13 (0.08)	0.70 (0.03)	4.27E-07
MLT1J	LTR	1.18 (0.04)	0.82 (0.03)	3.02E-15
MLT1K	LTR	1.19 (0.04)	0.95 (0.03)	1.34E-06
MLT1L	LTR	1.21 (0.06)	0.85 (0.03)	8.31E-08

Supplementary Table 19. Comparison of Expected (baseline-LD+blood chromatin) enrichment of TE in autoimmune traits and other traits. These are TE families/subfamilies that are significantly different enriched between autoimmune and other traits.

Annotation (X)	$\%\mathbf{X}_{\mathbf{ALL}}$	$\%\mathbf{X}_{\mathbf{ALLTE}}$	$\%\mathbf{X}_{\mathbf{ALLTE-unique}}$	$\%\mathbf{X}_{\mathbf{ALLTE-nonunique}}$
H3K27ac-PGC2	27.80	21.93	22.58	19.88
Transcribed-Hoffman	35.36	35.48	34.37	39.02
Repressed-Hoffman	44.92	47.58	48.29	45.33
Promoter-UCSC	4.91	3.08	3.00	3.32
PromoterFlanking-Hoffman	0.08	0.06	0.07	0.06
Intron-UCSC	39.80	36.10	35.69	37.40
H3K4me3-peaks-Trynka	4.41	2.63	3.04	1.33
H3K4me1-peaks-Trynka	17.86	11.83	13.72	5.83
FetalDHS-Trynka	9.00	4.01	5.18	0.41
Conserved-LindbladToh	3.01	0.33	0.43	0.01
DGF-ENCODE	14.25	9.75	11.79	3.24
DHS-peaks-Trynka	11.73	6.40	8.19	0.70
Enhancer-Andersson	0.45	0.26	0.31	0.10
H3K4me1-Trynka	44.03	33.50	36.81	22.98
H3K27ac-Hnisz	39.92	34.08	34.68	32.17
DHS-Trynka	17.60	10.15	12.85	1.54
H3K4me3-Trynka	13.99	9.89	10.92	6.63
TFBS-ENCODE	13.66	9.58	11.14	4.62
CTCF-Hoffman	2.42	1.86	1.90	1.73
Enhancer-Hoffman	4.40	2.79	3.10	1.83
H3K9ac-Trynka	13.26	7.79	8.31	6.17
SuperEnhancer-Hnisz	17.25	14.30	14.28	14.38
TSS-Hoffman	1.89	0.84	0.89	0.70
UTR-3-UCSC	1.38	0.49	0.50	0.47
UTR-5-UCSC	0.62	0.22	0.23	0.17

Supplementary Table 20. The depletion of functional annotations from the baseline-LD model in ALLTE SNPs is still observed when restricting to uniquely mappable ALLTE SNPs. Let ALLTE denote 1000 Genomes SNPs lying inside the union of all TE, ALLTE-unique denote ALLTE SNPs lying inside a 35-mer with mappability 1 (i.e. unique mappability) based on the ENCODE 35-mer track (ALLTE-unique) and ALLTE-nonunique denote other ALLTE SNPs. For each of the functional annotation X of the baseline-LD model (version 1.1), we report 4 quantities: the % of all SNPs that lie in X ($\%X_{\text{ALL}}$), the % of ALLTE SNPs that lie in X ($\%X_{\text{ALLTE}}$), the % of ALLTE-unique SNPs that lie in X ($\%X_{\text{ALLTE-unique}}$), and the % of ALLTE-nonunique SNPs that lie in X ($\%X_{\text{ALLTE-nonunique}}$) We observed that for certain functional annotation there exist a small difference between $\%X_{\text{ALLTE-unique}}$ and $\%X_{\text{ALLTE-nonunique}}$. However, for certain functional annotations this difference is significant. This result indicates that some of these annotations are not assayed as the same quality for repetitive region of the genomes as it is done for regions with uniquely mappability.

Annotation	Expected (%SNPs)	$\tau*(se)$	$\tau*$ (P)	Enrichment (se)	Enrichment (P)	Expected (baseline-LD)
ALLTE-unique	40.21	0.38 (0.06)	8.39E-11	0.93 (0.04)	3.19E-01	0.63 (0.01)
LINE-unique	17.11	0.24 (0.04)	2.43E-08	0.97 (0.06)	8.97E-01	0.41 (0.02)
SINE-unique	9.18	0.57 (0.06)	1.17E-18	1.96 (0.17)	1.86E-06	0.75 (0.02)
LTR-unique	8.52	0.18 (0.04)	2.30E-05	0.44 (0.11)	1.41E-04	0.46 (0.03)
DNA-unique	3.16	0.21 (0.06)	1.53E-04	1.40 (0.21)	1.96E-01	0.72 (0.01)

Supplementary Table 21. S-LDSC results for main annotations restricted to SNPs with mappability of 1. We report the heritability enrichment and $\tau*$ for four main TE classes (LINE, SINE, LTR, and DNA), and ALLTE restricted to the 85% of SNPs with mappability of 1; we refer to these annotations as LINE-unique, SINE-unique, LTR-unique, DNA-unique, and ALLTE-unique, respectively. Results are meta-analyzed across 41 independent traits. Results were very similar to analyses using all SNPs (Figure 1 and Supplementary Table 3).

Annotation	Expected (%SNPs)	$\tau*(se)$	$\tau*$ (P)	Enrichment (se)	Enrichment (P)
ALLTE-unique-age-match	34.08	0.37 (0.05)	2.95E-12	0.88 (0.04)	2.11E-01
LINE-unique-age-match	15.68	0.23 (0.04)	6.19E-08	0.71 (0.05)	6.68E-07
SINE-unique-age-match	7.42	0.55 (0.08)	1.09E-13	1.70 (0.25)	1.35E-03
LTR-unique-age-match	7.62	0.16 (0.04)	1.66E-04	0.39 (0.12)	1.04E-04
DNA-unique-age-match	3.01	0.20 (0.06)	7.19E-04	1.31 (0.22)	4.15E-01

Supplementary Table 22. S-LDSC results for main annotations restricted to SNPs with mappability of 1 and age-match between unique TE and original TE. We report the heritability enrichment and $\tau*$ for four classes (LINE-unique-age-match, SINE-unique-age-match, LTR-unique-age-match, DNA-unique-age-match), and ALLTE-unique-age-match. We constructed these annotations from LINE-unique, SINE-unique, LTR-unique, DNA-unique, and ALLTE-unique by considering families/subfamilies such that the distribution of age of these annotations match the original TE classes. Results are meta-analyzed across 41 independent traits. Results were very similar to analyses using all SNPs (Figure 1 and Supplementary Table 3).

Annotation	Expected (%SNPs)	$\tau*(se)$	$\tau*$ (P)	Enrichment (se)	Enrichment (P)
ALLTE-unique	47.08	0.31 (0.04)	6.92E-13	0.77 (0.02)	3.86E-09
LINE-unique	20.04	0.23 (0.05)	4.04E-05	0.82 (0.05)	1.72E-03
SINE-unique	10.75	0.59 (0.06)	1.57E-20	1.80 (0.14)	1.78E-06
LTR-unique	9.98	0.16 (0.04)	1.31E-05	0.39 (0.07)	8.81E-08
DNA-unique	3.71	0.20 (0.04)	4.61E-06	1.21 (0.15)	7.94E-01

Supplementary Table 23. S-LDSC results for main TE classes restricted to SNPs with mappability of 1, with reference panel SNPs also restricted to SNPs with mappability of 1. We report the heritability enrichment and $\tau*$ for four main TE classes (LINE-unique, SINE-unique, LTR-unique, and DNA-unique), and ALLTE-unique when only SNPs with mappability of 1 (85% of SNPs with mappability of 1) are Included in the 1000 Genomes reference panel used by S-LDSC to compute LD scores.. Results are meta-analyzed across 41 independent traits. Results were very similar to analyses using all SNPs (Figure 1 and Supplementary Table 3).

Annotation	Expected (%SNPs)	$\tau * (se)$	$\tau*$ (P)	Enrichment (se)	Enrichment (P)
ALLTE	53.25	0.33 (0.04)	1.17E-11	0.73 (0.03)	7.72E-01
LINE	21.19	0.19 (0.04)	3.85E-05	0.75 (0.05)	3.45E-06
SINE	16.14	0.44 (0.06)	7.42E-13	1.19 (0.11)	2.0E-01
LTR	9.86	0.13 (0.03)	3.68E-05	0.37 (0.07)	3.20E-08
DNA	3.28	0.14 (0.04)	8.63E-04	1.23 (0.19)	6.65E-01

Supplementary Table 24. S-LDSC results do not change significantly when removing human-specific TE. For all four TE classes (LINE, SINE, LTR, and DNA) and ALLTE, we removed the set of 14,870 human-specific TE identified by Tang et al.[18] (which is a superset of polymorphic TE). Results were very similar to analyses using all SNPs (Figure 1 and Supplementary Table 3).

Window	Repeat	$\tau * (se)$	$\tau*$ (P)	Enrichment (se)	Enrichment (P)
window-500bp	ALU	0.49 (0.06)	1.12E-15	1.18 (0.12)	2.35E-01
	DNA	0.15 (0.04)	8.64E-04	1.23 (0.19)	6.68E-01
	ERVK	0.09 (0.04)	1.88E-02	-0.66 (0.26)	3.50E-05
	L1	0.16 (0.04)	3.23E-04	0.62 (0.05)	2.18E-10
	LINE	0.18 (0.05)	1.73E-04	0.73 (0.05)	4.66E-07
	LTR	0.14 (0.03)	2.90E-05	0.38 (0.07)	2.81E-08
	ALLTE	0.31 (0.05)	1.14E-11	0.72 (0.03)	3.68E-12
	SINE	0.43 (0.06)	3.09E-12	1.18 (0.11)	2.73E-01
window-padding	ALU	0.30 (0.09)	5.49E-04	1.08 (0.14)	8.52E-01
	DNA	0.09 (0.06)	1.52E-01	1.32 (0.20)	4.13E-01
	ERVK	0.34 (0.10)	4.08E-04	-0.55 (0.30)	1.50E-03
	L1	-0.04 (0.09)	6.94E-01	0.65 (0.05)	5.56E-08
	LINE	0.03 (0.06)	5.78E-01	0.70 (0.05)	3.54E-07
	LTR	0.22 (0.06)	2.50E-04	0.47 (0.08)	2.33E-06
	ALLTE	0.17 (0.06)	1.02E-02	0.69 (0.03)	4.81E-13
	SINE	0.22 (0.08)	5.14E-03	1.04 (0.12)	8.23E-01

Supplementary Table 25. Choice of window size does not impact S-LDSC heritability enrichment results. In our main analysis, for each TE class/family we add an additional annotation that consist of a 500bp window around the TE. We refer to this annotation as window-500. To make sure our result is not biased to the window size (e.g., 500bp), we consider a model where we add four different window sizes (100, 200, 500, and 1000bp) around each TE annotation.

Supplementary Figures

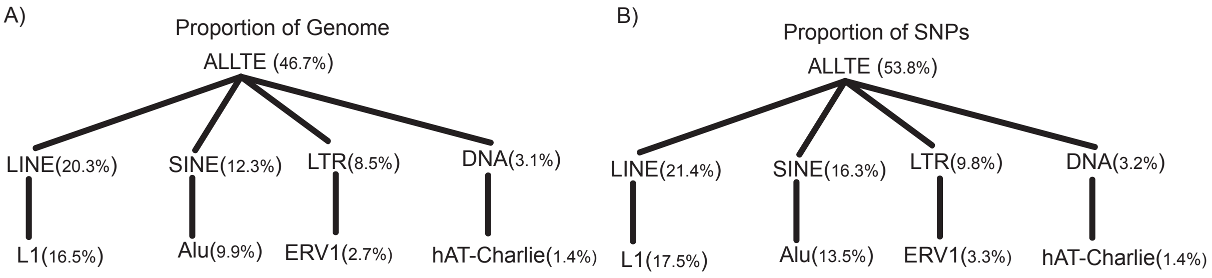

Supplementary Figure 1. Proportion of genome and proportion of SNPs spanned by each TE class and corresponding TE families. We have four main TE classes (LINE, SINE, DNA, and LTR). For each main TE class, we illustrated the family that captures the largest proportion of that TE class (L1 for LINE, Alu for SINE, hAT-Charlie for DNA, and ERV1 for LTR). (A) Proportion of genome that is captured by each TE. (B) Proportion of common SNPs that is captured by each TE.

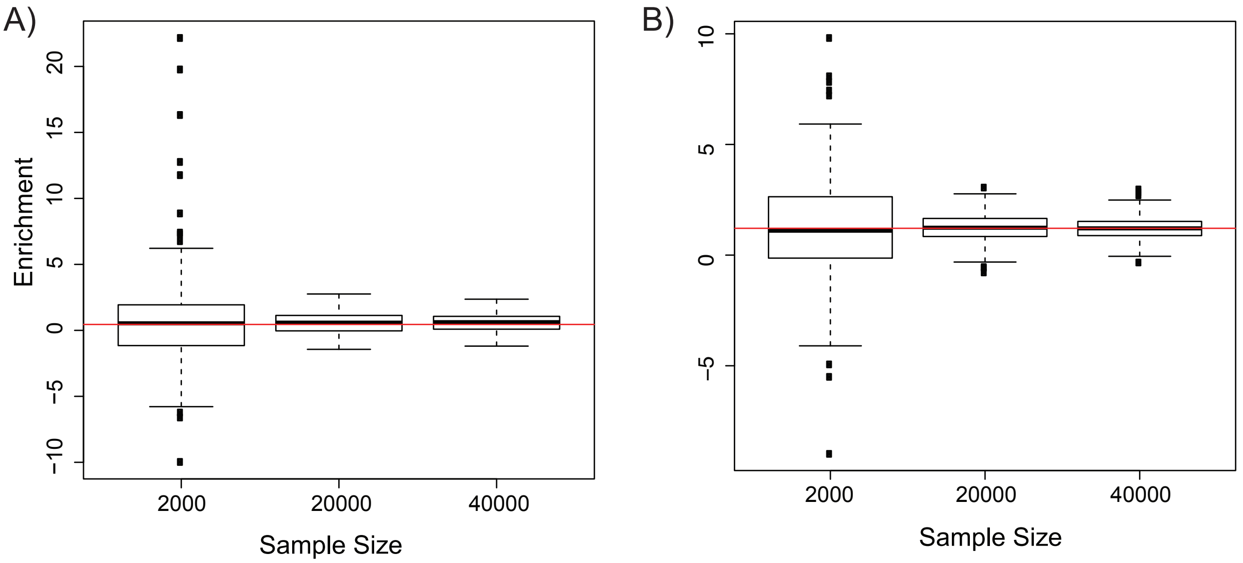

Supplementary Figure 2. S-LDSC produces unbiased estimates of enrichment in simulations with TE annotations. We simulated enrichment for different TE annotations. Then, we simulated the summary statistics for 2,000, 20,000, and 40,000 individuals where the genotypes are obtained from UK Biobank. After simulating the summary statistics, we applied S-LDSC conditional on baseline-LD model and the TE annotation. Regression SNPs in S-LDSC were obtained from the HapMap Project phase 3 [19] (see URLs). These SNPs are well-imputed SNPs. SNPs with marginal association statistics larger than 80 or larger than 0.001N and SNPs that are in the major histocompatibility complex (MHC) region were excluded from all the analyses. Reference SNPs were obtained using the European samples in 1000G [20]. Heritability SNPs, which are used to estimate h_g^2, were common variants (MAF≥0.05) in the set of reference SNPs. A) We simulated enrichment for LTR where we set LTR true enrichment value to be 0.44, which is close to the estimated enrichment for LTR in real dataset. B) We simulated enrichment for SINE where we set SINE true enrichment value to be 1.22, which is close to the estimated enrichment for SINE in real dataset. The red horizontal line indicates the true simulated enrichment for each TE. The X-axis is the number of individuals and the Y-axis is the estimated enrichment from S-LDSC.

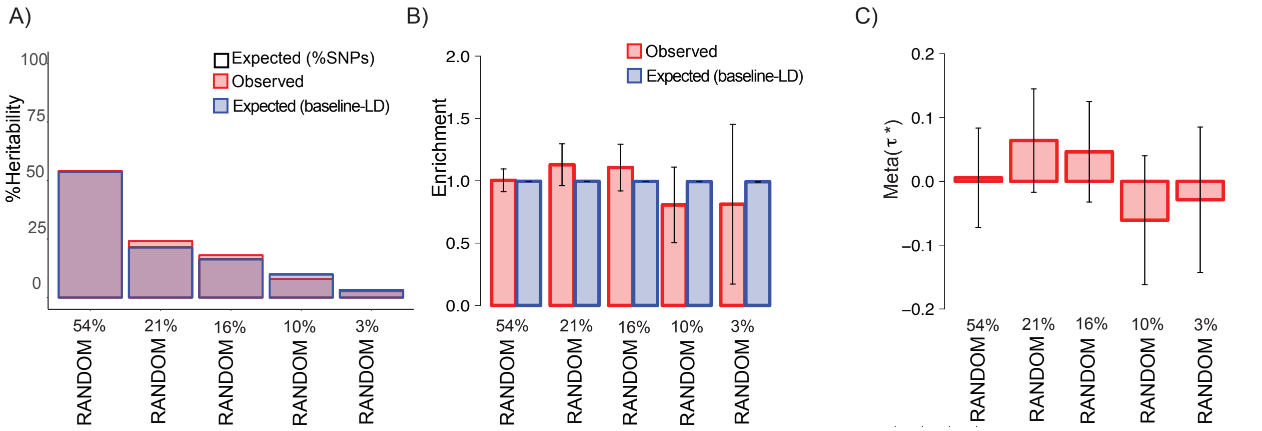

Supplementary Figure 3. Randomly selected control regions of the genome are not informative for disease heritability. For each of four main TE classes and ALLTE, we randomly selected control regions of the genome with the same %SNPs and we report A) three measures of %heritability: Expected (%SNPs), Observed, and Expected (baseline-LD); B) two measures of heritability enrichment: Observed and Expected (baseline-LD); and C) standardized effect size ($\tau*$), which quantifies effect that are unique to the focal annotation. Results are meta-analyzed across 41 independent traits. Numerical values of %SNPs are provided for each annotation. Error bars denote 95% confidence intervals.

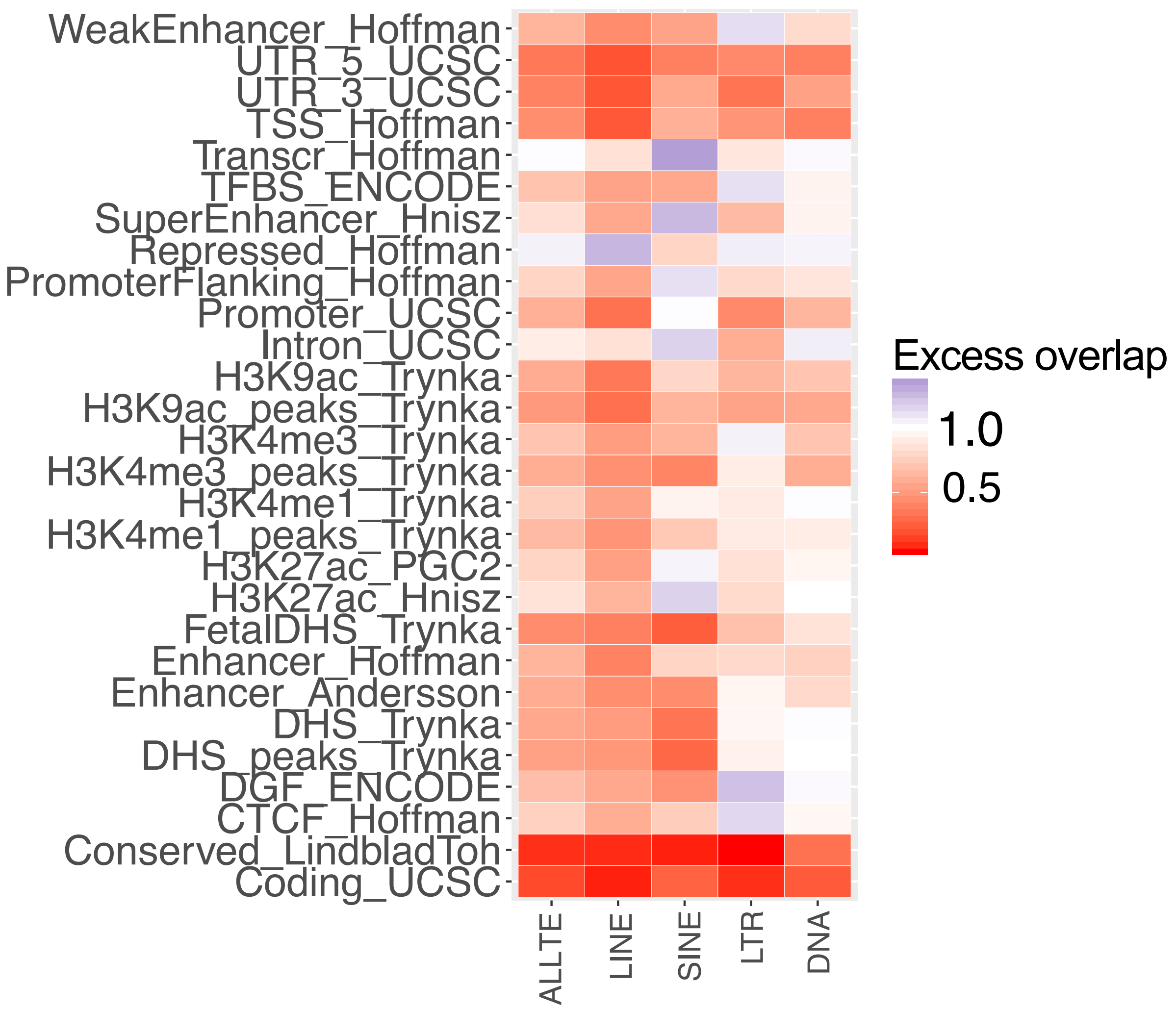

Supplementary Figure 4. Excess overlap between each TE class and functional annotations. The X-axis is the set of 5 TE (All, LINE, SINE, LTR, and DNA) and the Y-axis is the set of functional annotations in baseline model. We defined the excess overlap as the proportion of observed overlap between two annotations divide by the expected overlap between two annotations. Let A and B indicate two annotations and $|.|$ indicate the number of non-zero SNPs and we assume M is the total number of common SNPs. We defined the excess overlap as follows: Excess(A,B) $= \frac{\frac{|A \cap B|}{M}}{\frac{|A|}{M}\frac{|B|}{M}}$.

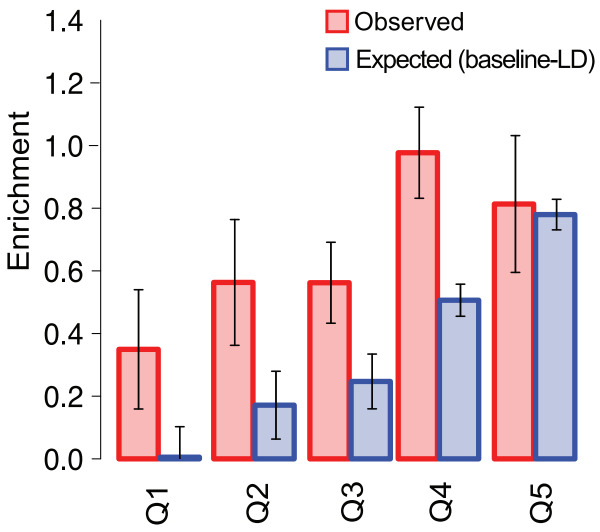

Supplementary Figure 5. S-LDSC results for each TE class in different age quintiles. We report the Observed and Expected (baseline-LD) enrichment of SNPs in each TE class for five different age quintiles where Q1 is the youngest and Q5 is the oldest. We computed the Observed enrichment conditional on ALLTE and the baseline-LD model. Error bars represent 95% confidence intervals. Numerical results are reported in Supplementary Table 6.

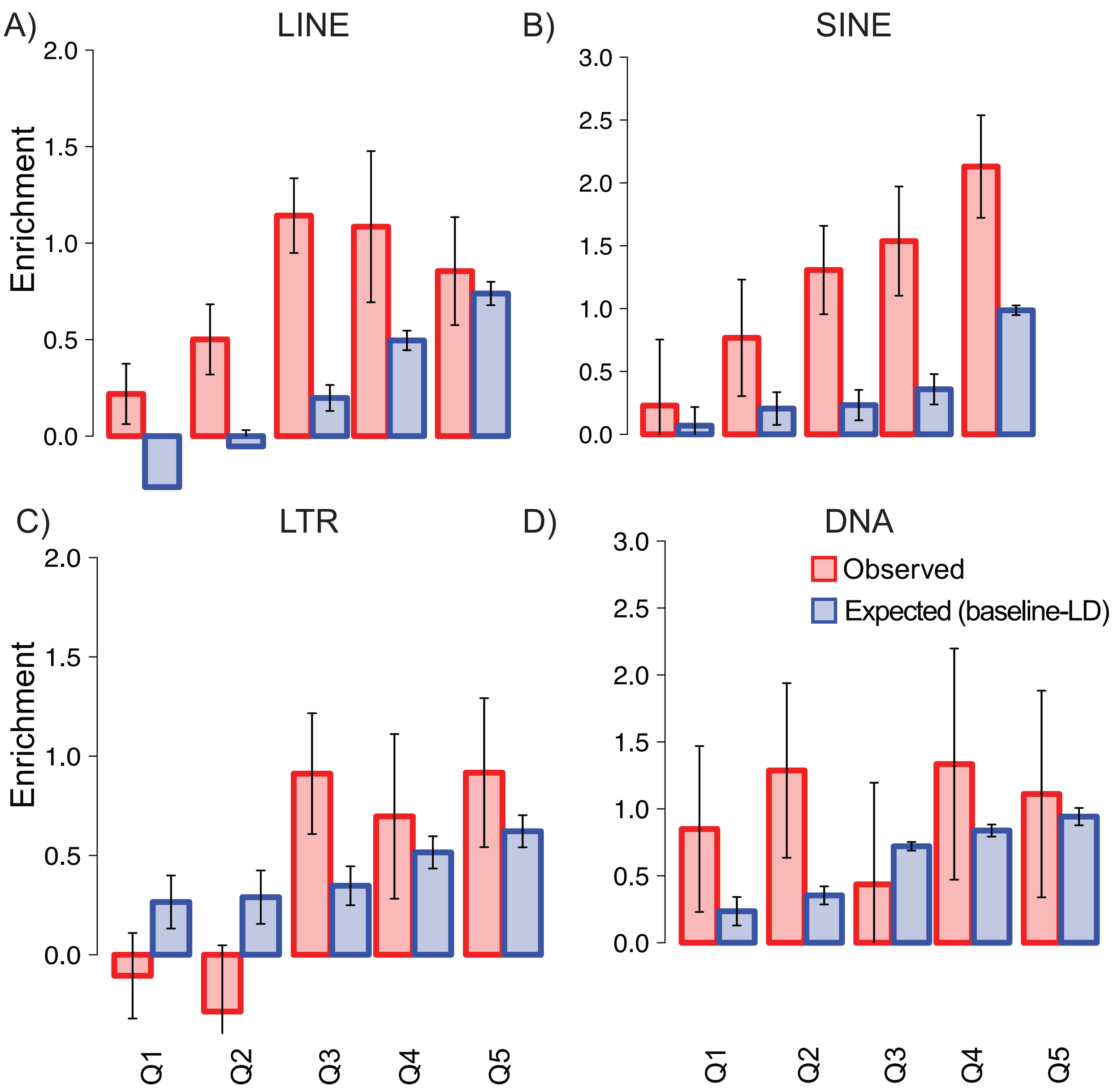

Supplementary Figure 6. S-LDSC results for all TE in different age quintiles. We report the Observed and Expected (baseline-LD) enrichment of SNPs in ALLTE for five different age quintiles where Q1 is the youngest and Q5 is the oldest. We computed the Observed enrichment conditional on ALLTE and the baseline-LD model. Error bars represent 95% confidence intervals.

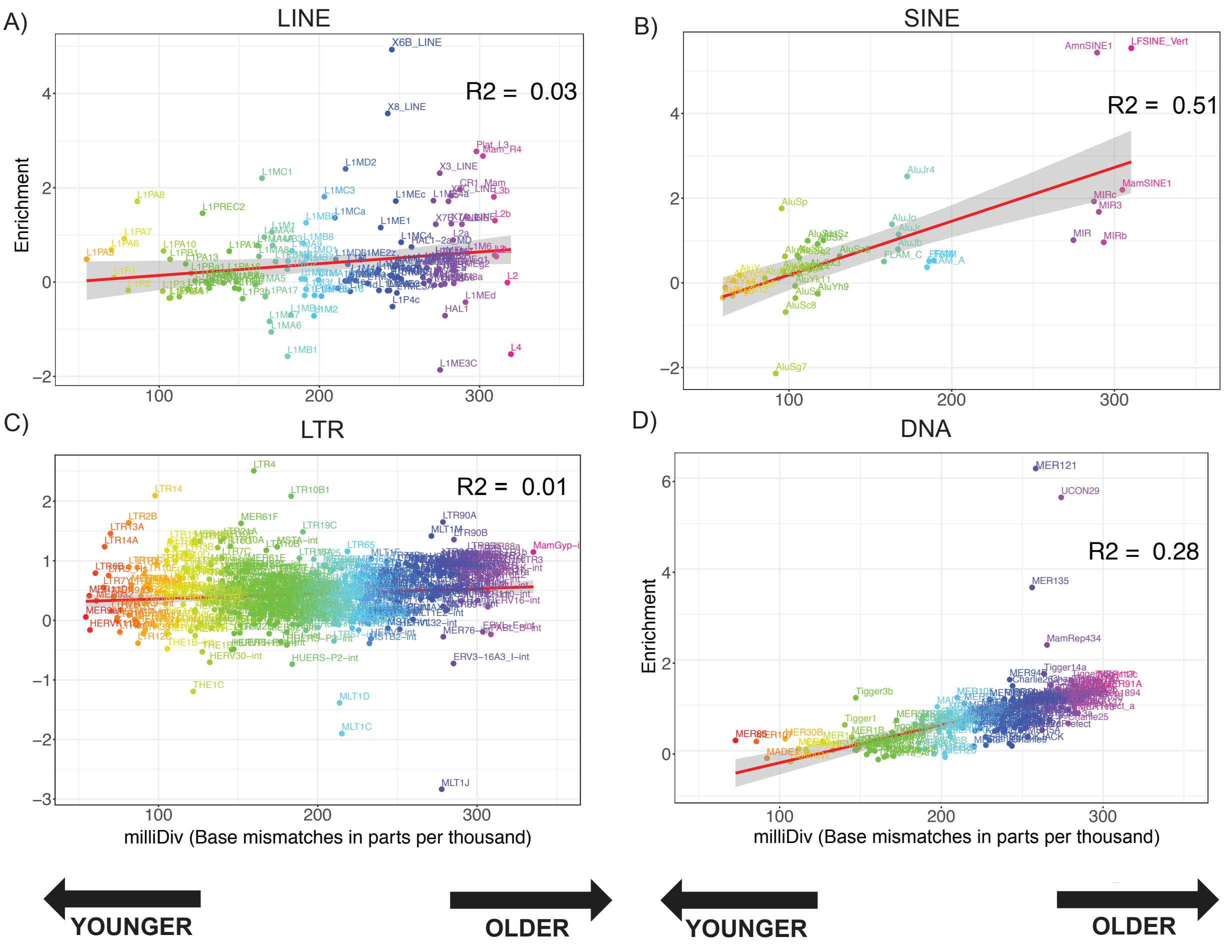

Supplementary Figure 7. S-LDSC results for individual TE families/subfamilies in each TE class as a function of age. Meta-analysis results of expected (baseline-LD) enrichment for (A) LINE, (B) SINE, (C) DNA, and (D) LTR across 41 traits. The X-axis is the age in miliDiv that is obtained from RepeatMasker software (see URLs). We use the miliDiv as an approximation of the age of TE. The Y-axis is the expected (baseline-LD) enrichment for each TE family/subfamily. The red line indicates a linear fit between the miliDiv and enrichment of each TE family/subfamily. This results are similar to what was observed in ref[21], who reported increased overlap with open chromatin regions for older TEs, although the baseline-LD model considers a broad range of coding, conserved, regulatory and LD-related annotations. There are significant outliers, especially for DNA transposons; for example, the MER121 subfamily has a Expected(baseline-LD) enrichment of 6.21$\pm$0.46. This implies that the MER121 subfamily is very strongly enriched for known functional elements. Several of the outliers, including MER121, were previously reported in ref[21] to have high overlap with open chromatin regions. Numerical results are reported in Supplementary Data 2.

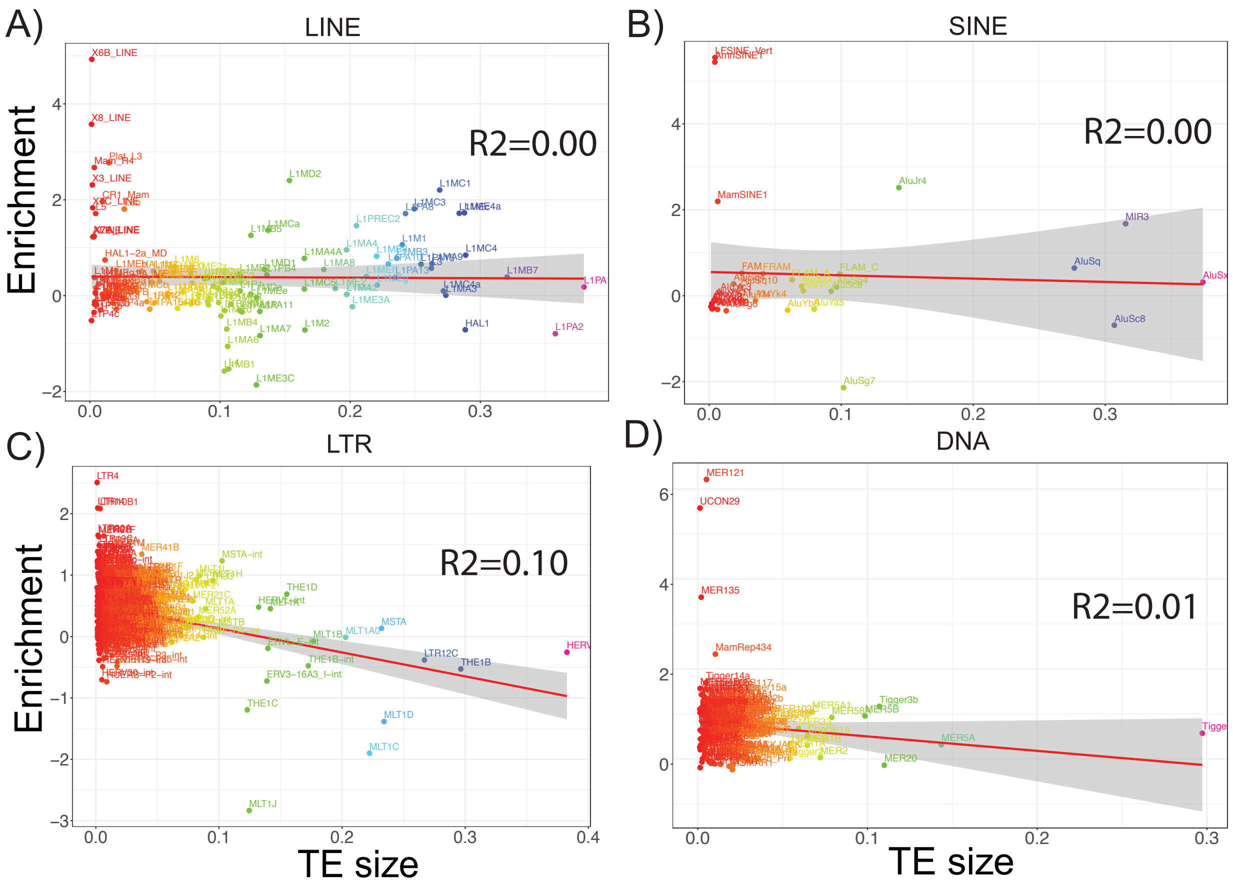

Supplementary Figure 8. S-LDSC results for individual TE families/subfamilies in each TE class as a function of TE size. Meta-analysis results of expected (baseline-LD) enrichment for (A) LINE, (B) SINE, (C) DNA, and (D) LTR across 41 traits. The X-axis is the TE size (%SNP). The Y-axis is the expected (baseline-LD) enrichment for each TE family/subfamily. The red line indicates a linear fit between the TE size and enrichment of each TE family/subfamily.

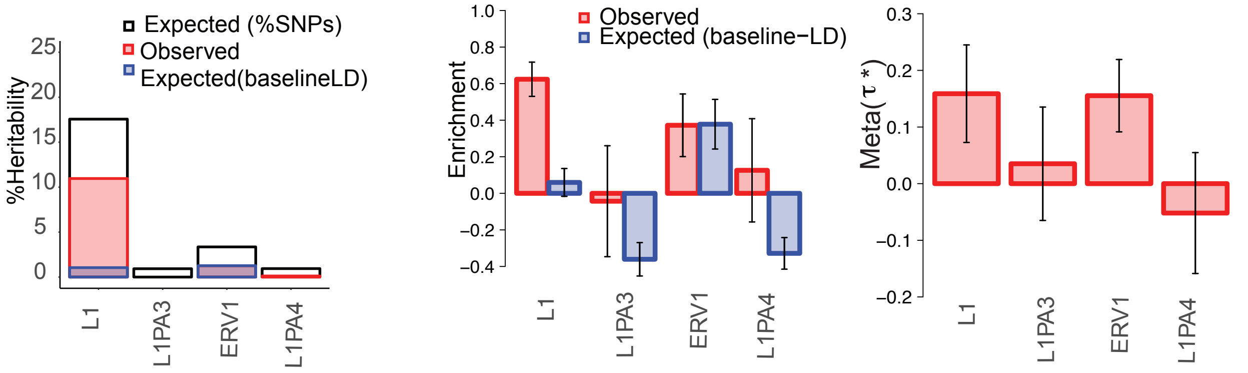

Supplementary Figure 9. TE families/subfamilies spanning at least 0.4% of common SNPs that are significantly depleted for disease heritability. A) Expected and observed %heritability (proportion of trait heritability) captured by each four TE families/subfamiles (L1, L1PA3, ERV1, L1PA4). Meta-analysis results across 41 traits of (B) enrichment and (C) $\tau*$ for all TE families/subfamiles that have significant enrichment conditional on the baseline-LD model. Error bars represent 95% confidence intervals. Numerical results are reported in Supplementary Table 8.

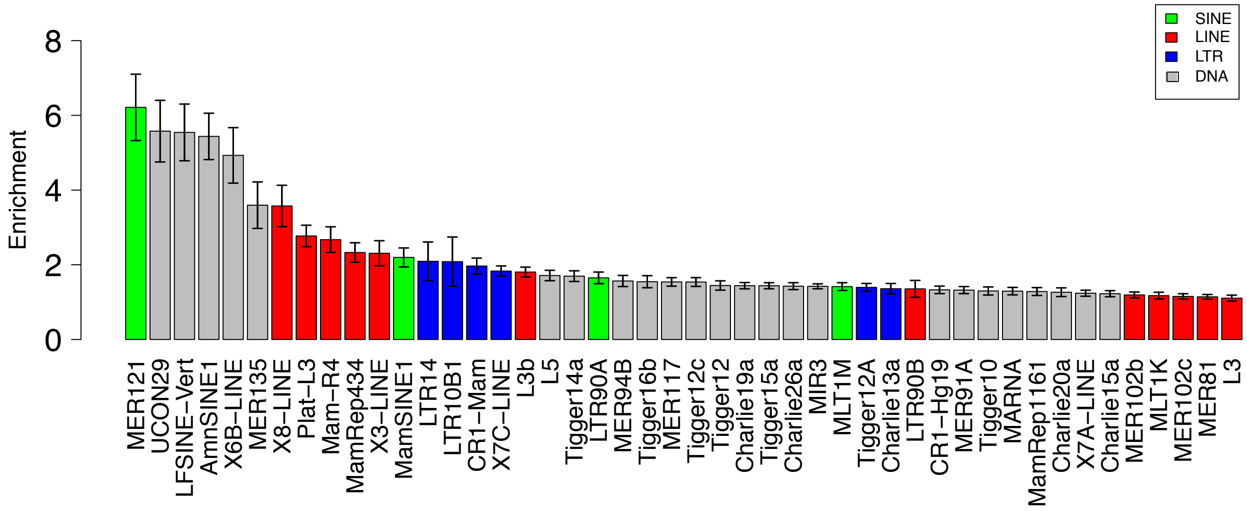

Supplementary Figure 10. TE families/subfamilies spanning less than 0.4% of common SNPs that are significantly enriched for disease heritability. We compute the trait enrichment of TE families/subfamilies that span less than 0.4% of SNPs by fitting a baselineLD model. Then we compute the per-SNP heritability based on τ estimated from baselineLD model. Enrichment of a TE is computed by summing over the per-SNP heritability of all SNPs that belong to TE. The X-axis is the set of TE and Y-axis is the trait enrichment. We detected 46 TE that are enriched for the heritability of disease and complex traits. Error bars represent 95% confidence intervals. Numerical results are reported in Supplementary Table 9.

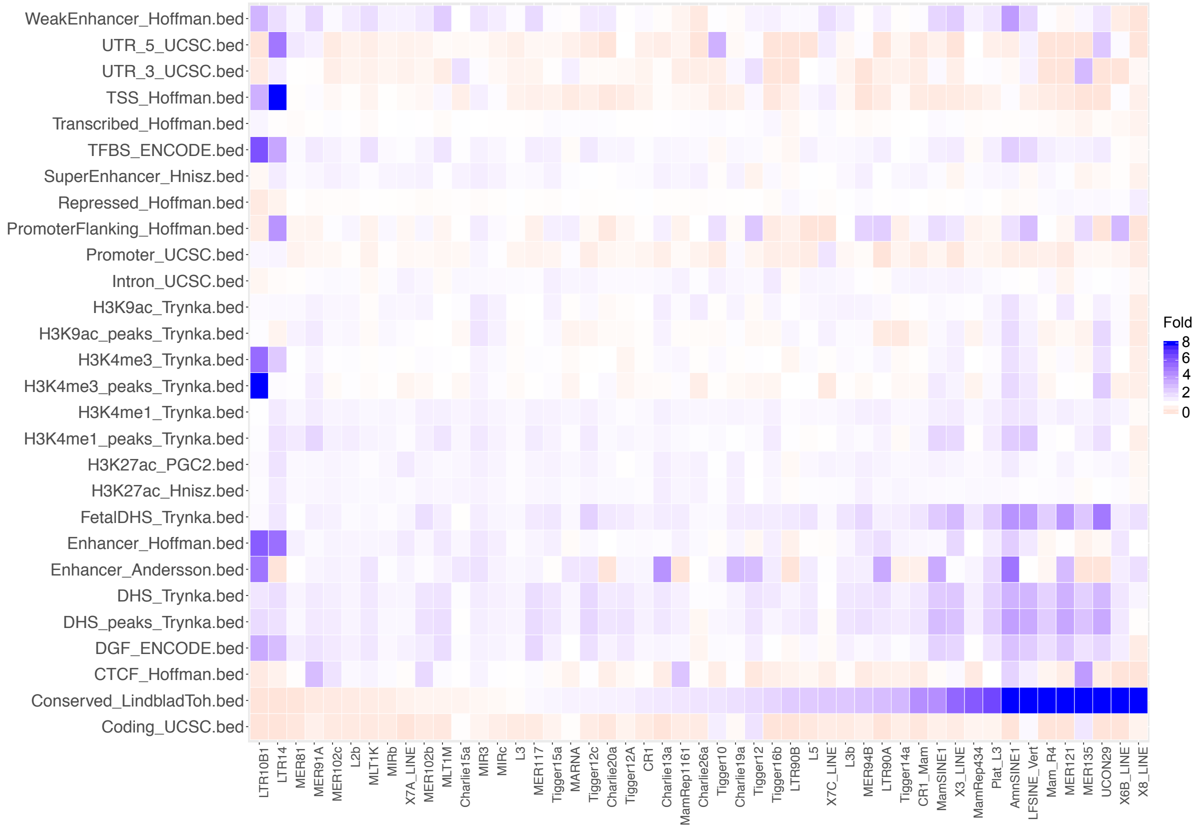

Supplementary Figure 11. Excess overlap between TE families/subfamilies spanning less than 0.4% of common SNPs that are significantly enriched for disease heritability and functional annotations. We only considered 46 TE that are enriched for trait heritability. The X-axis is the set of TE and the Y-axis is the set of functional annotations in baseline model. We defined the excess overlap as the proportion of observed overlap between two annotations divide by the expected overlap between two annotations. Let A and B indicate two annotations and $|.|$ indicate the number of non-zero SNPs and we assume M is the total number of common SNPs. We defined the excess overlap as follows: Excess(A,B) = $\frac{\frac{|A\cap B|}{M}}{\frac{|A|}{M}\frac{|B|}{M}}$. We compute the standard error over our estimates using block jackknife with 200 blocks (see Methods). Numerical results are reported in Supplementary Data 6.

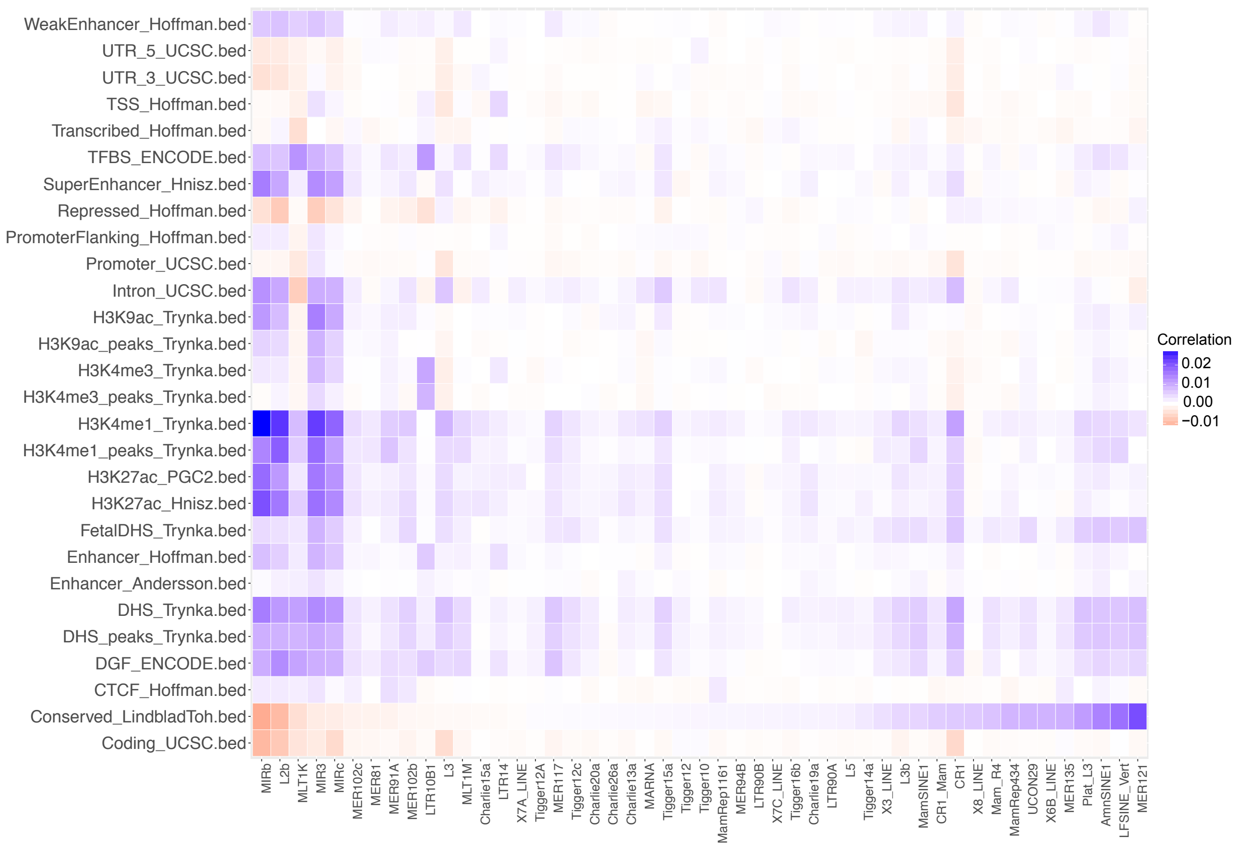

Supplementary Figure 12. Correlation between TE families/subfamilies spanning less than 0.4% of common SNPs that are significantly enriched for disease heritability and functional annotations. We only considered 46 TE that are enriched for trait heritability. We use correlation as a measure to compute similarity between two annotations. We compute the correlation between each TE and each baseline annotation. We compute the standard error over our estimates using block jackknife with 200 blocks (see Methods). We sort the TE in an increasing order of conserved annotation correlation with each TE. Numerical results are reported in Supplementary Data 7.

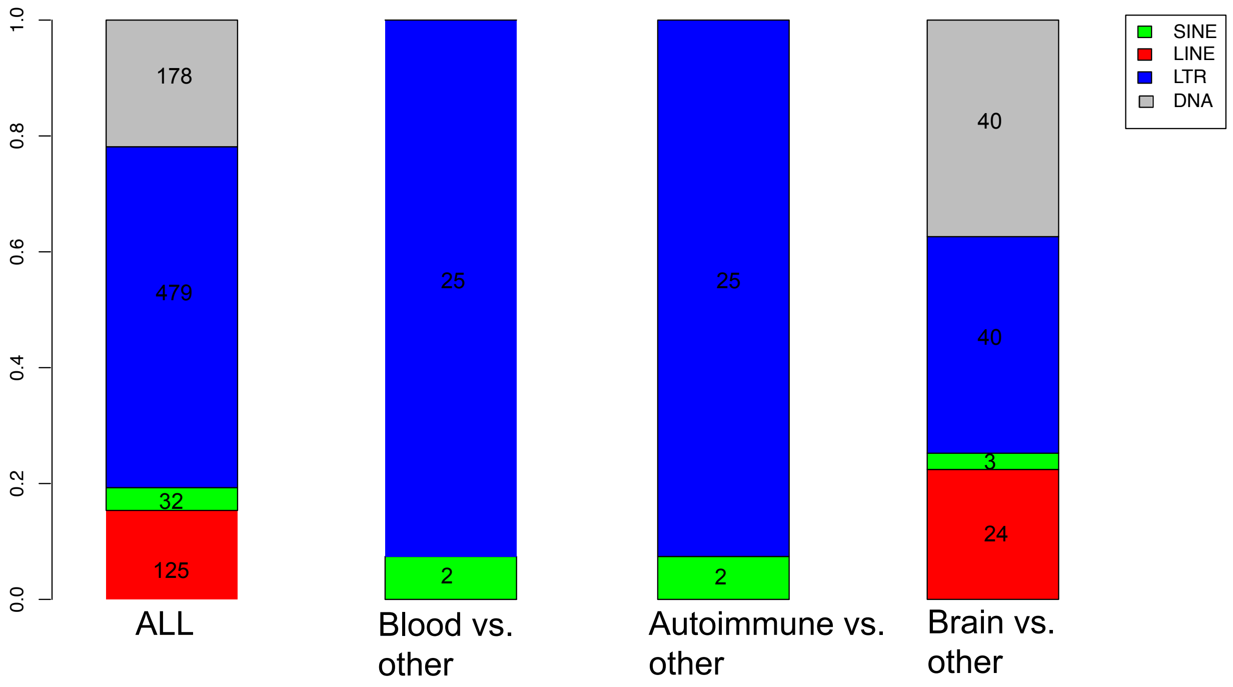

Supplementary Figure 13. Distribution of TE classes for TE families/subfamilies with trait class-specific Expected enrichment. We report the distribution of TE classes for (first bar) all 814 TE families/subfamilies spanning less than 0.4% of common SNPs, (second bar) the subset of 27 TE families/subfamilies with significantly higher Expected (baseline-LD+blood chromatin) enrichment for blood-related traits vs. other traits, (third bar) the subset of 27 TE families/subfamilies with significantly higher Expected (baseline-LD+blood chromatin) enrichment for autoimmune diseases vs. other traits, and (fourth bar) the subset of109 TE families/subfamilies with significantly higher Expected (baseline-LD+brain chromatin) enrichment for brain-related traits vs. other traits.

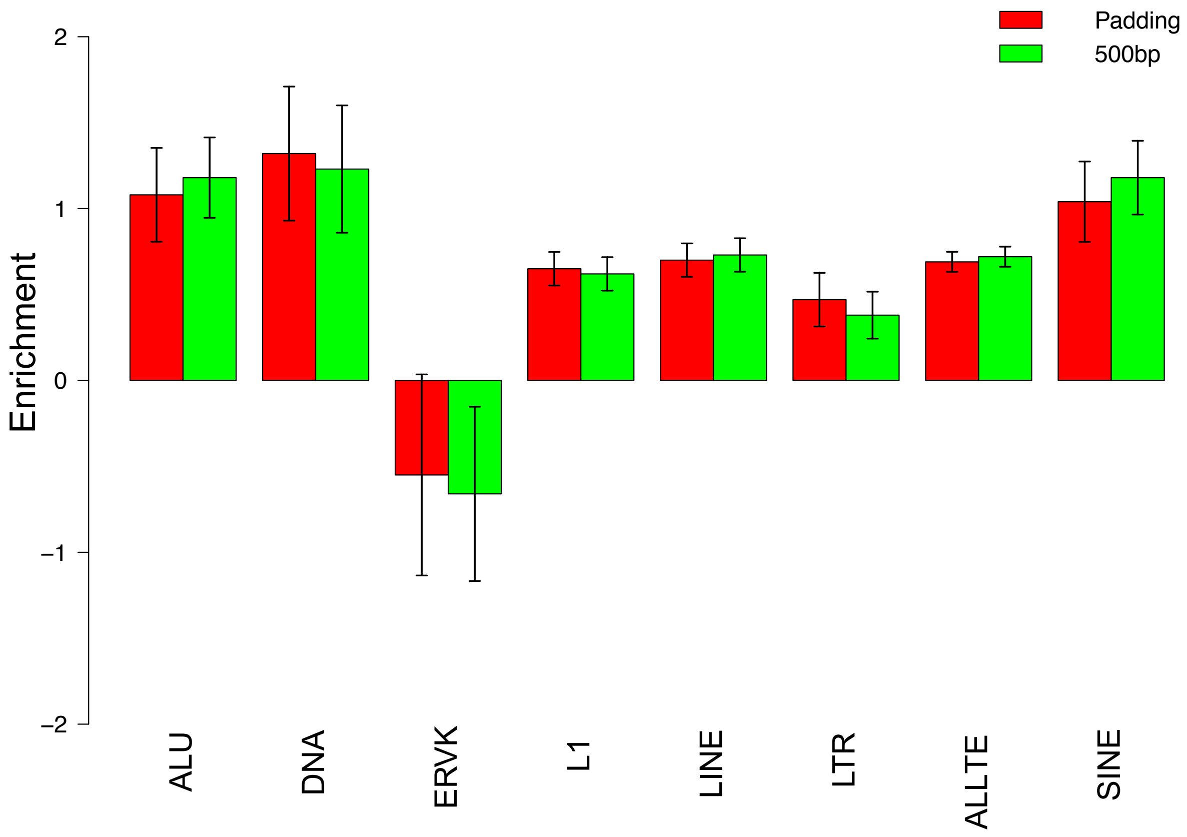

Supplementary Figure 14. Comparing S-LDSC enrichment of TE classes/families for different window sizes. In our main analysis, for each TE we add an additional annotation that consist of a 500bp window around the TE. We refer to this annotation as window-500. To make sure our result is not biased to the window size (e.g., 500bp), we consider a model where we add four different window sizes (100, 200, 500, and 1000bp) around each TE annotation. We refer to this result as window-padding. Error bars represent 95% confidence intervals. Numerical results are reported in Supplementary Table 25.

References

1. Finucane, H. K. *et al.* Partitioning heritability by functional annotation using genome-wide association summary statistics. *Nature Genetics* **47**, 1228–1235 (2015).

2. Consortium, E. P. An integrated encyclopedia of dna elements in the human genome. *Nature* **489**, 57–74 (2012).

3. Hoffman, M. M. *et al.* Integrative annotation of chromatin elements from encode data. *Nucleic Acids Research* **93**, 779–797 (2012).

4. Kundaje, A. *et al.* Integrative analysis of 111 reference human epigenomes. *Nature* **518**, 317–330 (2015).

5. Trynka, G. *et al.* Chromatin marks identify critical cell types for fine mapping complex trait variants. *Nature Genetics* **45**, 124–130 (2012).

6. Hnisz, D. *et al.* Super-enhancers in the control of cell identity and disease. *Cell* **155**, 934–947 (2013).

7. Lindblad-Toh, K. *et al.* A high-resolution map of human evolutionary constraint using 29 mammals. *Nature* **478**, 476–482 (2011).

8. Andersson, R. *et al.* An atlas of active enhancers across human cell types and tissues. *Nature* **507**, 455–461 (2014).

9. Davydov, E. V. *et al.* Identifying a high fraction of the human genome to be under selective constraint using gerp++. *PLoS Computational Biology* **6**, e1001025 (2010).

10. Gazal, S. *et al.* Linkage disequilibrium–dependent architecture of human complex traits shows action of negative selection. *Nature Genetics* **49**, 1421–1427 (2017).

11. Loh, P.-R. *et al.* Contrasting genetic architectures of schizophrenia and other complex diseases using fast variance-components analysis. *Nature Genetics* **47**, 1385–1392 (2015).

12. Loh, P.-R., Kichaev, G., Gazal, S., Schoech, A. P. & Price, A. L. Mixed-model association for biobank-scale datasets. *Nature Genetics* **50**, 906–908 (2018).

13. Hormozdiari, F. *et al.* Leveraging molecular quantitative trait loci to understand the genetic architecture of diseases and complex traits. *Nature Genetics* **50**, 1041–1047 (2018).

14. Farh, K. K.-H. *et al.* Genetic and epigenetic fine mapping of causal autoimmune disease variants. *Nature* **518**, 337–343 (2014).

15. Huang, H. *et al.* Fine-mapping inflammatory bowel disease loci to single-variant resolution. *Nature* **547**, 173–178 (2017).

16. Ernst, J. & Kellis, M. Large-scale imputation of epigenomic datasets for systematic annotation of diverse human tissues. *Nature Biotechnology* **33**, 364–376 (2015).

17. Hach, F. *et al.* mrsFAST-ultra: a compact, SNP-aware mapper for high performance sequencing applications. *Nucleic Acids Research* **42**, W494–W500 (2014).

18. Tang, W., Mun, S., Joshi, A., Han, K. & Liang, P. Mobile elements contribute to the uniqueness of human genome with 15, 000 human-specific insertions and 14 mbp sequence increase. *DNA Research* **25**, 521–533 (2018).

19. Integrating common and rare genetic variation in diverse human populations. *Nature* **467**, 52–58 (2010).

20. A map of human genome variation from population-scale sequencing. *Nature* **467**, 1061–1073 (2010).

21. Jacques, P.-É., Jeyakani, J. & Bourque, G. The majority of primate-specific regulatory sequences are derived from transposable elements. *PLoS Genetics* **9**, e1003504 (2013).
