# Supplementary material for: Functional disease architectures reveal unique biological role of transposable elements
Source: Nat Commun. 2019 Sep 6;10:4054. doi: 10.1038/s41467-019-11957-5 (PMC6731302; doi:10.1038/s41467-019-11957-5)
Supplement: Supplementary file 4 — Description of Additional Supplementary Files [file 41467_2019_11957_MOESM4_ESM.pdf]

**Supplementary Data 1. List of 47 data sets analyzed in this work**. We obtained publicly available summary statistics from previous published studies. UK Biobank summary statistics were previously computed using BOLT-LMM. For some traits we have more than one data set, thus we have 41 independent traits. However, we utilized all 47 data sets in our meta-analyses as the number of samples that overlap is low. These traits were selected based on a heritability z-score > 6. The 41 traits and 47 data sets are identical to those analyzed in Hormozdiari2018 et al.

**Supplementary Data 2. S-LDSC results for 854 TE classes/families/subfamilies.** We computed the Expected (baseline-LD) 853 TE that capture at least 0.001% of common SNPs. These results are obtained by meta-analyzing 41 independent traits and disease. We fit the baselineLD model for each trait. Then, using S-LDSC model we can estimate the per SNP heritability (variance of each SNP). We compute the heritability of each TE by summing over the per SNP heritability of all SNPs in each corresponding TE. The miliDiv column is obtained from RepeatMasker software (see URLs). We use the miliDiv as an approximation of the age of the repeat.

**Supplementary Data 3. List of all 854 TE classes/families/subfamilies spanning at least 0.001% of common SNPs obtained from RepeatMasker software.** It is worth mentioning that we have 853 TE and one ALLTE. ALLTE is the combination of all TE. Thus, we have 854 rows in this file.

**Supplementary Data 4. S-LDSC results for 814 TE families/subfamilies spanning less than 0.4% of common SNPs.** We computed the Expected (baseline-LD) 814 TE that capture at least 0.001% of common SNPs and less than 0.4%. These results are obtained by meta-analyzing 41 independent traits and disease. We fit the baselineLD model for each trait. Then, using S-LDSC model we can estimate the per SNP heritability (variance of each SNP). We compute the heritability of each TE by summing over the per SNP heritability of all SNPs in each corresponding TE. The miliDiv column is obtained from RepeatMasker software (see URLs). We use the miliDiv as an approximation of the age of the repeat.

**Supplementary Data 5. Set of TE families/subfamilies that are depleted for the Expected (baseline-LD) heritability of disease and complex traits.**

**Supplementary Data 6. Excess overlap of 854 TE classes/families/subfamilies and functional annotations.** We compute the excess overlap between all the 853 TE classes/families/subfamilies and the baseline-LD functional annotations. We defined the excess overlap as the proportion of observed overlap between two annotations divide by the expected overlap between two annotations. Let A and B indicate two annotations and |.| indicate the number of non-zero SNPs and we assume M is the total number of common SNPs. We defined the excess overlap as follows: $Excess(A,B) = \frac{|A \cap B|/M}{|A|/M\,|B|/M}$. We compute the standard error over our estimates using block jackknife with 200 blocks (see Methods).

**Supplementary Data 7. Correlation of 854 TE classes/families/subfamilies with baseline-LD model annotations.** We compute the correlation overlap between all the 853 TE classes/families/subfamilies and the baseline-LD functional annotations. We compute the standard error over our estimates using block jackknife with 200 blocks (see Methods).

**Supplementary Data 8. Comparison of Expected (baseline-LD+brain chromatin) enrichment of TE in brain-related traits and other traits.** These are TE families/subfamilies that are significantly different enriched between brain-related traits and other traits
